# Correction of Monogenic and Common Retinal Disorders with Gene Therapy

**DOI:** 10.3390/genes8020053

**Published:** 2017-01-27

**Authors:** Jesse D. Sengillo, Sally Justus, Thiago Cabral, Stephen H. Tsang

**Affiliations:** 1 Jonas Children’s Vision Care, and Bernard & Shirlee Brown Glaucoma Laboratory, Department of Ophthalmology, Columbia University Medical Center, New York, NY 10032, USA; js3899@cumc.columbia.edu (J.D.S.); sj2750@cumc.columbia.edu (S.J.); tc2803@cumc.columbia.edu (T.C.); 2 Edward S. Harkness Eye Institute, New York-Presbyterian Hospital, New York, NY 10032, USA; 3 State University of New York Downstate Medical Center, Brooklyn, NY 11203, USA; 4 Department of Ophthalmology, Federal University of Espírito Santo, Vitória 29075-910, Brazil; 5 Department of Ophthalmology, Federal University of São Paulo, São Paulo 04021-001, Brazil; 6 Department of Pathology & Cell Biology, Institute of Human Nutrition, College of Physicians and Surgeons, Columbia University, New York, NY 10032, USA

**Keywords:** gene therapy, genome engineering, CRISPR, retinal degeneration, inherited retinal disease

## Abstract

The past decade has seen major advances in gene-based therapies, many of which show promise for translation to human disease. At the forefront of research in this field is ocular disease, as the eye lends itself to gene-based interventions due to its accessibility, relatively immune-privileged status, and ability to be non-invasively monitored. A landmark study in 2001 demonstrating successful gene therapy in a large-animal model for Leber congenital amaurosis set the stage for translation of these strategies from the bench to the bedside. Multiple clinical trials have since initiated for various retinal diseases, and further improvements in gene therapy techniques have engendered optimism for alleviating inherited blinding disorders. This article provides an overview of gene-based strategies for retinal disease, current clinical trials that engage these strategies, and the latest techniques in genome engineering, which could serve as the next frontline of therapeutic interventions.

## 1. Introduction

Gene therapy holds the potential to revolutionize physicians’ ability to apply precision medicine. Depending on the inheritance pattern of a given genetic defect, gene therapy may involve the delivery of genetic material that either silences a pathogenic mutation or supplements patients with a wild type copy of a mutated gene. Enthusiasm for such strategies grew quickly, until the death of a clinical trial patient following a gene therapy-based intervention for ornithine transcarbamylase (OTC) deficiency [1], in addition to other adverse outcomes following gene therapy [2], prompted careful reevaluation of the ethical and safety considerations involved with such strategies. Vector choice and delivery strategies came under close scrutiny, and additional testing in animal models was emphasized as an essential pre-clinical safety measure.

In 1996, a team that included Drs. Bennett, Maguire and Gouras presented the first study to use gene transfer to rescue retinal degeneration in a mouse model of retinitis pigmentosa (RP) [3]. Subretinal injection of a recombinant adenovirus carrying wild type cDNA of the phosphodiesterase β subunit (*βPDE*) effectively delayed photoreceptor degeneration in the *rd* mouse model, which harbors biallelic mutations in the *β* subunit of rod cGMP-phosphodiesterase. Later, in 2001, injection of an adeno-associated virus (AAV) delivering subretinal *RPE65* to an *RPE65^−/−^* canine model was reported to delay disease progression and even restore visual function [4]. These studies provided an initial preclinical proof-of-principle for such strategies to be translated to clinical trials. Multiple groups subsequently launched clinical trials to deliver subretinal *RPE65* via an AAV vector to patients with Leber congenital amaurosis [5,6,7], reinvigorating research in gene therapy.

The eye has been at the forefront of developments in gene-based technology because it possesses several unique anatomical and physiological properties. The eye and retina are easily accessible to physicians, and in clinical trials, the untreated eye can serve as a control if the condition progresses symmetrically [8,9]. The optical transparency of the eye allows in vivo, longitudinal, and non-invasive monitoring of disease progression and treatment response [10]. Moreover, the retina can be viewed with impressively high resolution, on the scale of microns, with spectral domain optical coherence tomography (SD-OCT), enabling visualization and delineation of the individual layers of the retina [10,11]. Retinal imaging continues to improve, and OCT imaging can now be acquired *en face*, with images oriented parallel to the plane of the retina as opposed to perpendicular, as in the traditional SD-OCT system [11,12,13,14,15,16]. These improvements can lead to an even deeper understanding of previously well-characterized diseases and open new doors for less-studied conditions. OCT, as well as other imaging modalities such as fundus autofluorescence, have allowed investigators to characterize retinal disease and identify potential outcome measures for disease progression and treatment efficacy, supplementing traditional tests of visual function [10,17,18,19,20,21,22,23,24,25,26,27]. Another factor that makes the eye more approachable to gene therapy is its relatively immune-privileged status. This is maintained by a lack of lymphatics, immunomodulatory factors in the vitreous humor, and antigen-presenting cells, hence leading to a significantly weaker immune response to novel antigens [28].

The advantages the eye confers for studying gene-based therapies has accelerated research initiatives in this area, and today, there are multiple clinical trials utilizing such strategies in the treatment of retinal disease (Table 1). This article will review some of the parameters that must be considered for gene delivery in the eye, provide examples of applications of these techniques in current clinical trials, and present an overview of some of the latest advances in genome surgery and their future applications to treating inherited retinal dystrophies.

## 2. Vector Choice

While there exist a variety of techniques for delivering genes into target cells, the most popular options for use in patients include viral-mediated strategies typically involving one of two viruses: AAVs or lentiviruses. AAV, a single-stranded DNA vector belonging to the family *Parvoviridae*, remains the vector of choice for most ocular gene therapy applications due to several advantageous properties: AAVs elicit less immunogenic reactions than that observed from administration of adenoviral vectors; they exhibit minimal self-replication; and they can last for up to six years after a single administration [76,77]. Furthermore, a recombinant AAV (rAAV) capsid can be selected or altered to suit different experimental needs and to optimize transfection [78,79,80]. While each of these characteristics are advantageous for gene-based therapy, some challenges remain.

A known limitation of AAVs is their carrying capacity, which is approximately 4.5 kb, making gene-based delivery difficult for longer genes [81]. Researchers studying delivery of genes larger than 5 kb have circumvented this issue by utilizing dual AAV strategies. Yan et al. were the first to employ a dual AAV strategy, effectively delivering the erythropoietin genomic locus to mouse muscle tissue [82]. A similar study reported that this dual AAV technique could be applied to the retina [83], and subsequent studies successfully delivered the *MYO7A* gene in vitro and in vivo. Encouragingly, co-infected cells showed recombination of the two halves, leading to a full-length gene product [84,85]. Another strategy for delivering large genes is with lentiviral vectors. Lentiviruses are retroviruses that can carry up to 8 kb of transgene material [86]. Large genes such as *MYO7A* (Usher syndrome) and *ABCA4* (Stargardt disease) have been approached successfully with lentiviruses in pre-clinical studies [64,67] and are currently being tested in clinical trials (Table 1).

## 3. Vector Delivery

### 3.1. Subretinal Injection

Most gene therapy trials for retinal degenerations involve subretinal injection of the vector-containing therapeutic (Table 1). To accomplish this, a transient retinal detachment is made intraoperatively to create a subretinal space to place the injection. This detachment resolves spontaneously [9]. The viral vector subsequently occupies the subretinal space and infects target cells of the neurosensory retina or retinal pigment epithelium (RPE). Subretinal injections are particularly localized, which has been postulated to affect transfection and may explain the variable patterns of functional vision improvement that were observed in the clinical trials of Leber congenital amaurosis (LCA) [41,87]. For example, portions of the detachment that reattach first (such as the edges) may have lower transfection [41,87,88]. Although there is no proven superior alternative to subretinal injection for most retinal degenerations at this time, patients may benefit from more homogeneously distributed and less invasive strategies [87].

### 3.2. Intravitreal Injection

The intravitreal AAV2 virus that is currently used predominantly infects the ganglion cell layer that lies close to the vitreous cavity [89,90]. This is in comparison to subretinal injections, in which cells in the area of the induced bleb, such as photoreceptors and RPE, are transduced [91]. For therapies targeting the inner retina, such as in Leber hereditary optic neuropathy (LHON), the intravitreal route of administration may prove successful in clinical trials (Table 1). The intravitreal approach is also less invasive, thus potentially decreasing the chance for complications [92]. This may be advantageous for retinas that are particularly at risk for retinal detachment, such as in X-linked retinoschisis (XLRS), which is caused by mutations in retinoschisin 1 (*RS1*) [93]. Furthermore, RS1 protein is normally secreted and may impart its effects on areas that are more distant to that which is transduced by a subretinal injection [93,94]. Although the eye is considered to be immune-privileged, previous experiments in mice suggest that the vitreous compartment may respond differently to AAV2 compared to the subretinal space [95,96]. Li et al. found that intravitreal injection of AAV2-CBA-PEDF resulted in a humoral immune response which prevented therapeutic effects upon readministration in a mouse model for choroidal neovascularization [96]. The same effect was not observed via subretinal administration. In LHON and XLRS pre-clinical studies, however, favorable safety and biodistribution were reported for single injections of AAV-containing gene therapy administered to the vitreous, corroborating findings in phase I trials for LHON that report no inflammatory response of the vitreous or anterior chamber (Table 1) [54,56,57,58]. Biodistribution of gene-based therapeutics delivered to the vitreous remains complex, but pre-clinical and phase 1 clinical trial results are encouraging.

### 3.3. Non-Viral Strategies

To date, non-viral gene delivery strategies have not been used in human retina, but they deserve mention due to their success and continued improvements in animal models. Electroporation is a strategy developed by Matsuda and Cepko [97] in which DNA is directly injected in the subretinal space without being packaged into a vector. A non-invasive, high voltage pulse is subsequently administered, leading to changes in membrane potential and permeability, and thus cellular uptake of the nucleic acid [98]. This method is especially useful for quickly testing promoter activity and the effect of protein overexpression in models of retinal degeneration [97]. Previously thought to have little therapeutic potential in humans, electroporation has advanced significantly, particularly through suprachoroidal injection of DNA followed by ab externo electroporation, which is less invasive and results in a large area of transfection [99].

Nanoparticles (NPs) have also been studied for the purpose of gene therapy delivery, mainly through polymers, liposomes, and peptide-compacted DNA, which has been extensively reviewed elsewhere [100,101]. Compared to AAV technology, NPs may be more cost-effective but have lower transfection efficiency [102,103]. Additional advantages of NPs over AAVs include a carrying capacity up to 20 kbp and a lower likelihood of complications, as there is less potential for insertional mutagenesis. This technology, like electroporation, has not been translated to clinical trials but has shown progress as an approach to ocular gene therapy [104,105,106,107,108,109,110,111,112].

## 4. Update on Gene Therapy Trials for Retinal Disease

### 4.1. Leber Congenital Amaurosis

LCA causes a severe early-onset retinal dystrophy with an estimated prevalence of 2–3 per 100,000 births [113]. As previously stated, gene therapy trials for *RPE65*-associated LCA are arguably among the major accomplishments in modern medicine (Table 1). RPE65 is a protein required for recycling visual pigment in the retinal pigment epithelium [114]. Mutations in *RPE65* are one cause of LCA, which typically manifests as severe visual dysfunction at birth with a pigmented retina, wandering nystagmus, and amaurotic pupils [115]. After efficacy was shown in a naturally occurring canine model, multiple clinical trials confirmed that an AAV vector delivering *RPE65* was safe and improved visual function during the follow-up of LCA patients [4,5,6,7,29,31,32,33,40,116,117,118,119]. Spark Therapeutics (Philadelphia, PA, USA) is sponsoring a phase III trial for bilateral subretinal delivery of AAV containing *RPE65*. In an October 2015 press release, Spark stated the trial met its primary endpoint of improved bilateral mobility test change score and two secondary endpoints, which included favorable full-field light sensitivity and mobility test change scores for the first treated eye compared to baseline. Visual acuity was a secondary outcome measure, but it was not met.

### 4.2. Choroideremia

Approximately 1 in 50,000 individuals are affected by choroideremia (CHM), an X-linked retinal dystrophy [120]. Affected males typically present with night blindness and a progressively constricting visual field, similar to retinitis pigmentosa [121]. A mutation in *CHM*, which expresses REP-1, leads to chorioretinal degeneration starting in the mid-periphery. A pale fundus is observed as the sclera illuminates through the degenerated layers of RPE and choriocapillaries. Female carriers may exhibit changes on electroretinography despite no visual complaints [120,122]. Multiple phase I/II studies to test the efficacy of rAAV-REP1 delivery are ongoing (Table 1). Initial findings reported by MacLaren et al. [43] suggested improved visual function in two of six patients, even in the context of iatrogenic retinal detachment that is required intraoperatively (NCT01461213). Increased sensitivity measured via microperimetry in the treated eyes correlated with vector dose per area of live retina. A follow-up study showed sustained improvement in visual acuity of the same two patients after 3.5 years, while the control eyes continued to progress [123]. Choroideremia patients possibly have less surgical risk when treated earlier, as one treated patient had intraoperative complications from extensive retinal detachment. Hence, treatment may be more appropriate when there is less cell loss and a more intact retinal structure [124].

### 4.3. Achromatopsia

Patients with achromatopsia, also known as rod monochromatism, typically experience decreased visual acuity, nystagmus, photophobia, decreased color vision, and a central visual field defect. The condition is estimated to affect 1 in 30,000 individuals [125,126]. On OCT, an optical gap can be seen, representing foveal hypoplasia [126]. This condition is inherited recessively, and multiple genes of the phototransduction pathway have been implicated. Most commonly, the cyclic nucleotide-gated ion channel B3 gene (*CNGB3*) is mutated in patients of European descent [127]. *CNGB3* encodes a subunit of the CNG channel, which closes in the presence of a light stimulus, hyperpolarizing the photoreceptor [128]. Applied Genetic Technologies Corporation (AGTC) (Alachua, FL, USA) is sponsoring a phase I/II trial delivering rAAV2tYF-PR1.7-hCNGB3 to achromatopsia patients (Table 1). Additionally, another phase I/II gene therapy clinical trial has launched in Tübingen, Germany for patients with achromatopsia caused by defects in a similar gene, *CNGA3* (Table 1).

### 4.4. X-Linked Retinoschisis

XLRS is a common cause of macular degeneration in young males and is estimated to affect between 1 in 5000 and 1 in 25,000 individuals [129]. Characteristic symptoms include central vision loss and mildly decreased visual acuity due to foveomacular schisis. A spoke-wheel appearance encompassing the fovea is seen on fundoscopy, and OCT reveals cysts throughout the macula, including the fovea [130]. Two phase I/II clinical trials sponsored by the National Eye Institute (NEI) and AGTC are ongoing (Table 1). Each trial is testing therapeutic delivery of the causative gene, *RS1*, utilizing a different vector and promoter. Both trials are delivering the vector via an intravitreal injection, which is likely more ideal, as the retina in XLRS patients is fragile, and the foveomacular schisis does not provide ideal anatomy for subretinal injection [94]. Retina specialists should carefully assess XLRS patients for enrollment in clinical trials, as patients’ visual function may not be sufficiently reduced to meet inclusion criteria.

### 4.5. Leber Hereditary Optic Neuropathy

LHON is an inherited mitochondrial disease that causes degeneration of the retinal ganglion cells and is thus characterized by optic atrophy. The prevalence is estimated to be approximately 1 in 31,000 to 50,000 among Northern European populations, and the three most common genetic causes are mutations in *ND1*, *ND4*, or *ND6*, which code for a key enzyme in cellular respiration in mitochondria [131]. Current vectors of gene therapy cannot faithfully deliver therapeutic genes directly into the mitochondria. Researchers overcame this challenge by demonstrating that allotopically expressed *ND4* delivered into the vitreous via an AAV vector was effective in a mouse model of LHON [132,133,134]. This strategy has subsequently been translated to a clinical trial sponsored by GenSight Biologics (Paris, France). Initial results from the open-label phase I study reported no outstanding adverse events following administration of GS010, an AAV vector carrying normal *ND4*, in patients with *ND4*-associated LHON. At the 90-day follow-up appointment, two patients had an increase in visual acuity, and three patients were unchanged from baseline [58]. In the phase III study, which is currently recruiting subjects, the effects of intravitreal GS010 injection will be compared to a sham-control.

### 4.6. Retinitis Pigmentosa

RP is a genetically heterogenous disease with nearly 80 genes associated with the condition, which can be inherited in multiple fashions, namely dominantly, recessively, or in X-linked or mitochondrial forms [135]. Affecting approximately 1 in 4000 individuals, RP presents at various ages with night blindness as a first symptom. Degeneration typically starts at the mid-periphery and progresses towards the macula, corresponding to a constricting visual field [136]. Homozygous or compound heterozygous mutations in the mer receptor tyrosine kinase (*MERTK*) gene are a rare cause of autosomal recessive RP, which is being approached with gene therapy at the King Khaled Eye Specialist Hospital and King Faisal Specialist Hospital & Research Center (Riyadh, Saudi Arabia). Results of a phase I trial administering rAAV2-VMD2-hMERTK to one eye in each of six patients with *MERTK*-associated RP were recently reported [62]. Safety of the vector was confirmed, and three patients experienced a post-surgical increase in visual acuity; however, this was transient in two of the patients, whose improvements returned to baseline by the two-year follow-up appointment. Another trial for clinically-diagnosed RP patients (mutation- and gene-independent) is assessing the efficacy of RST-001, an intravitreally administered gene therapy that expresses a photoswitch, channelrhodopsin-2, which allows transfected cells of the inner retina to respond to light even after photoreceptors have degenerated (Table 1). Proof-of-principle studies in mice and marmosets supported the initiation of a phase I/II clinical trial sponsored by Retrosense Therapeutics (Ann Arbor, MI, USA) [60,61].

### 4.7. Usher Syndrome

Syndromic retinitis pigmentosa associated with neurosensory hearing loss, Usher syndrome (USH), occurs with a prevalence of approximately 3–6 per 100,000 individuals, although this may be an underestimate [137,138]. In the absence of genetic testing, Usher syndrome can be diagnosed clinically if irreversible hearing loss occurs in the setting of retinitis pigmentosa and a family history that suggests an autosomal recessive inheritance pattern. Usher syndrome type 1 (USH1) is characterized by profound hearing loss at birth, early-onset retinitis pigmentosa, and possibly decreased vestibular function. Mutations in myosin 7a (*MYO7A*) can cause USH1 and are currently being studied in gene therapy clinical trials. Normally, MYO7A is involved in protein and organelle transport across photoreceptor cilia [139]. Because *MYO7A* is large and difficult to package in AAV, the drug UshStat^®^ was developed by Sanofi (Paris, France) to deliver the therapeutic gene in a lentivirus vector [64]. Phase II of this trial is projected to finish in 2018.

### 4.8. Stargardt Disease

Affecting approximately 1 in 10,000 individuals, Stargardt disease is an inherited macular degeneration associated with dysfunction in the ATP-binding cassette, subfamily A, member 4 (*ABCA4*) gene [140]. Bi-allelic pathogenic mutation of *ABCA4* causes accumulation of toxic byproducts in the RPE, which subsequently leads to cell degeneration [140]. Sanofi is studying the efficacy of SAR422459, a lentiviral vector providing addition of the normally functioning gene (Table 1). Completion of phase II is anticipated in 2017. In the meantime, Parker et al. have defined criteria for statistically significant changes in functional and structural measurements in Stargardt disease progression [65].

### 4.9. Neovascular Age-Related Macular Degeneration

Gene therapy has also been applied to acquired retinal disease (Table 1). Age-related macular degeneration (AMD) is the leading cause of visual impairment in the United States, and the neovascular form, or wet AMD (wAMD), is characterized by abnormal choroidal vessel proliferation behind the macula [68,141]. Over-secretion of vascular endothelial growth factor (VEGF) plays a key role in the pathogenesis of neovascular AMD, and the current standard of care includes the administration of intravitreal anti-VEGF inhibitors [142]. Gene therapy has a theoretical advantage, as long-lasting expression would reduce or eliminate the need for repeated injections of anti-VEGF inhibitors in patients. Lions Eye Institute (Perth, WA, Australia) is sponsoring a trial for subretinal delivery of rAAV.sFLT-1, and no serious ocular or systemic adverse events due to the vector were reported in phase I/IIa trials [68,70]. Two patients experienced mild eye and anterior chamber inflammation suspected to have been caused by rAAV.sFLT-1 administration, although the inflammation did resolve. While the cohort receiving gene therapy required fewer additional ranibizumab injections, post-hoc analysis revealed no significant differences in outcome measures between groups in the phase 2a study. This may be in part due to the low number of patients enrolled (*n* = 32), and future trials would be necessary to determine if rAAV.sFLT-1 is an efficacious adjunct to ranibizumab [70]. Similarly, Genzyme, a Sanofi company (Cambridge, MA, USA) is sponsoring a phase I safety and tolerability trial assessing intravitreal delivery of the same anti-VEGF gene therapy vector. Oxford BioMedica (Oxford, UK) has also initiated clinical trials for subretinal RetinoStat^®^ (GEM study), a lentivirus containing angiostatic genes, angiostatin and endostatin, to prevent vessel growth and subsequent vision decline (Table 1). Campochiaro et al. reported results of the GEM study, which showed vector-containing gene therapy delivered by subretinal injection was well tolerated, and expression levels were maintained at last measurement, which was more than 2.5 years in eight subjects and more than 4 years in two subjects [75]. It remains to be seen if gene therapy will provide a viable treatment option and/or adjunct for wAMD. If efficacy is demonstrated, this may represent a paradigm shift in ophthalmic medicine, as gene therapy has traditionally been applied to monogenic diseases [70].

## 5. Importance of Natural Disease Characterization

The advent of clinical trials for inherited retinal disease has necessitated natural disease history studies for various degenerations. Although gene therapy trials use the contralateral eye as a control, it is important to study the rate of disease progression and the degree of symmetry between each eye to validate using the untreated eye as a control. Assessing disease progression rates from large cohort studies is additionally useful for identifying optimal functional and structural outcome measures [10]. Moreover, results from these studies aid investigators in deciding which patients are the best candidates for gene therapy. Although it can be difficult to complete a well-powered study given the low sample size of patients with rare degenerative diseases, several observational cohort studies are being attempted for a variety of retinal degenerations (Table 2).

## 6. CRISPR/Cas: Ophthalmic Genome Surgery of the Future?

Clustered regularly interspaced short palindromic repeats (CRISPR) are present in bacterial and archaeal genomes and function to provide immunity against invading viruses [143,144,145,146]. The CRISPR locus contains integrated pieces of foreign DNA and encodes for a long transcript that is ultimately processed intracellularly to become CRISPR-derived RNA (crRNA). Consequently, crRNAs contain complementary sequences to the previously invading foreign DNA, and they can mediate a highly specific, nucleic-acid based adaptive immune system by complexing with CRISPR-associated (Cas) nucleases that induce double strand breaks (DSBs) [147]. Also within the CRISPR locus of a strain of *Streptococcus pyogenes*, there exists a necessary noncoding *trans*-activating crRNA (tracrRNA), which investigators combined with crRNA to form a chimeric crRNA-tracrRNA hybrid, termed guide RNA (gRNA) [148]. Cas9 was shown to effectively be directed to specific target sequences by custom-made gRNA in vitro, introducing a new genome-modifying toolkit which has revolutionized the field of genetic engineering [148].

Once a double-strand break occurs in the eukaryotic genome, either non-homologous end joining (NHEJ) or homology-directed repair (HDR) subsequently occurs to prevent cell death. NHEJ occurs with less fidelity, as the two broken ends are directly reconnected with the possibility of insertions and deletions, termed ‘indels’, often yielding a dysfunctional gene product. In the case of HDR, the homologous chromosome serves as a template to ensure the broken strands contain the correct sequence before ligation [149]. DSBs caused by the CRISPRn/Cas system (where ‘n’ denotes traditional CRISPR, namely to distinguish it from newer techniques, which will be discussed in subsequent text) at a desired location induce these endogenous DNA repair mechanisms. Thus, applications of CRISPRn/Cas have made use of the NHEJ and HDR that occurs after induction of DSBs. NHEJ occurs more frequently compared to HDR, making the gene ‘knock-out’ strategy more readily feasible thus far [150]. However, there is also ‘knock-in’ strategies that supplement a donor template in addition to the gRNA and Cas protein, which consequently promotes gene repair via the HDR pathway [151]. Previous genomic engineering also required HDR to occur; however, these methods were dependent on the low incidence of DNA damage, whereas CRISPRn creates DSBs with impressive specificity [152].

### 6.1. Applications to Retinal Disease

Retinal disease has been at the forefront of previous gene-based therapy research and, for the same reasons, may prove to be amenable to CRISPRn/Cas in vivo applications. Of note, Bakondi et al. used CRISPRn/Cas9 to ablate the mutant rhodopsin gene containing an *S334ter* mutation in a rat model of dominant retinitis pigmentosa. Sub-retinal delivery of gRNA and Cas9 with subsequent electroporation was sufficient to ablate the gain-of-function mutation and proved highly specific, as function of the wild type allele was unaffected. Phenotypic rescue was evident with immunohistology, showing robust photoreceptor and retinal synapse preservation [153]. This study marked the first instance where a dominantly inherited mutant allele was amenable to ablation through CRISPRn/Cas in vivo. In addition to this rescue experiment, CRISPRn/Cas has already been used to validate disease-causing pathogenic gene mutations in the retina [154,155] and identify genes important for normal retinal function and development [156,157,158].

Repurposing the prokaryotic CRISPRn/Cas adaptive immune system to perform gene-editing in human cells has also been accomplished in vitro [159,160,161,162], representing a major milestone in molecular biology [150]. For example, induced pluripotent stem cells (iPSCs) obtained from skin fibroblasts of a patient with X-linked retinitis pigmentosa caused by an *RPGR* point mutation were subjected to CRISPRn/Cas9 gene editing, producing corrected graft cells [163]. In theory, these corrected iPSCs can be used for autologous cell transplantation, which will circumvent the need for immunosuppression. For mature retinal cells, in vivo delivery experiments have been done with AAV2, which provided Cas9 and sgRNA that targeted the yellow fluorescent protein (*YFP*) gene expressed in a transgenic mouse [164]. This strategy effectively decreased the number of cells expressing YFP in the retina without affecting retinal function, suggesting that applying CRISPRn/Cas to the mature retina in vivo is feasible.

Despite rapid developments in CRISPR/Cas genome engineering, various challenges persist. Off-target effects remain a concern for future therapeutic applications, as mutagenesis of non-desired sites can occur at a high frequency [165]. To circumvent this issue, a catalytic domain of Cas9 was reengineered in such a way that the nuclease could function as a nickase, which induces a single strand break (SSB) that, in the event of an off-targeting event, will likely be less deleterious (Cas9n) [166,167,168]. To obtain a DSB at the desired location using Cas9n, two gRNAs are used that complement opposite strands of the target sequence. This improves specificity by more than an order of magnitude [166,167,168]. Another study optimized the reduction of off-target effects in human cells, characterizing the effects of base pair mismatch location and number in the gRNA, and the advantages of titrating the dosage of Cas9 and gRNA [169]. Delivery poses an additional challenge for the clinical translation of CRISPR/Cas to human disease. While the most frequently used Cas protein has a cDNA sequence that can be packaged into AAV, its small carrying capacity does not allow much additional room for other elements, as in the case of gene ‘knock-in’ where a donor template is required.

### 6.2. New Developments in CRISPR-Based Genome Engineering

While CRISPR in its traditional form has become a staple of most genetic labs, its utility has significantly diversified as researchers began developing ways to repurpose the technology. Recently, a strategy for reversible CRISPR-based gene editing was developed and termed CRISPR interference (CRISPRi). This technique employs a catalytically dead Cas (dCas) protein, which can bind but not cleave DNA [170]. Thus, the dCas–sgRNA complex binds to the target gene and impedes transcription by preventing RNA polymerase from binding to the promoter region or from elongating a transcript [170]. In microbial organisms like *Escherichia coli*, simply replacing Cas with dCas is typically sufficient to prevent transcription, but in mammalian cells, the efficiency of inhibition is significantly enhanced by fusing the dCas with a repressor domain, the Krüppel-associated box (KRAB) domain being one commonly used repressor [171]. This arrangement creates a highly programmable system that can alter gene expression at the transcription level. The technique holds many similarities to the conventionally used RNA interference (RNAi) system, but it has been shown to be highly sensitive to mismatches between the sgRNA and target sequence, thus reducing off-targeting effects compared to RNAi [172,173].

While KRAB domains can be fused with dCas, other molecules have been added to the endonuclease to alter its activity in the opposite fashion. By fusing transcription activating domains with dCas, gene expression can be enhanced rather than inhibited. This technique is known as CRISPR-on or more commonly, CRISPR activation (CRISPRa) [174], and it typically involves fusion of dCas with transactivator domain VP16, among others [175,176,177]. By localizing the dCas to the promoter region of a gene, VP16 can upregulate the gene’s expression. While only minimal activation has been obtained using this strategy, designing multiple sgRNAs for the same promoter region has been shown to significantly augment efficiency [178]. Efforts are underway to further enhance the efficiency of activation by recruiting additional activation domains, for example, and there remains great enthusiasm for the future applications of this technique.

One of the newest developments in CRISPR arose from researchers seeking a means to create single-nucleotide alterations without inducing DSBs [179,180]. They did so by fusing dCas with a rodent cytidine deaminase called APOBEC1, which converts cytosine to uracil. Cells transfected with the new dCas/deaminase fusion protein undergo a single cytosine to uracil conversion, which is subsequently corrected to thymine by DNA correction mechanisms or during replication. Komor et al. created several generations of the fusion protein to optimize its efficiency, and they dubbed the final generation BE3. The optimized BE3 protein replaces dCas with a nickase, which, as mentioned previously, is a catalytically active Cas protein that induces only a SSB. Although dCas results in the fewest number of insertions and deletions (indels) in the genome due to the lack of a cleavage event, nickase-induced SSBs also result in much fewer indels compared to the number accrued following a DSB. The rationale for using a nickase over dCas was that by nicking the non-edited homologous strand, the cell perceives the strand to be newly synthesized, which signals for DNA repair proteins to correct it for errors using the edited target strand as a template. In this manner, both strands will be edited despite only one having been cleaved. BE3 editing efficiency was further enhanced by fusing Cas nickase with an inhibitor of the naturally occurring base excision repair process, which removes uracil nucleotides from DNA. The overall efficiency of the system combined with the reduction in the frequency of indels and off-targeting effects represent a considerable advancement and evolution from the traditional application of CRISPR.

When juxtaposing all four techniques, CRISPRi and CRISPRa distinguish themselves from traditional CRISPRn/Cas or BE3 approaches because they act at the transcriptome level, preserving the original genome. This allows the techniques to be reversible, which confers both benefits and detriments. The transient effects of CRISPRi/a impart a considerable advantage over CRISPRn in obtaining FDA approval for use in clinical trials, since the patient’s own genome will remain intact and unaltered. However, for patients with inherited retinal conditions, for example, multiple subretinal injections to re-administer the Cas/sgRNA-containing vectors could be harmful and impractical. A long-term, single intervention would be preferable for these patients, in which case CRISPRn or the BE3 system might be a stronger alternative. Additionally, despite the many advantages of the BE3 technology over traditional CRISPRn/Cas, such as reduced off-targeting effects and higher overall efficiency of editing, the current BE3 strategy is limited in its applicability. Until conversions can be made between adenine and guanine as well, the breadth of diseases that can be corrected using this technique is restricted to a select subset. However, CRISPR technology has continued to advance at a rapid pace, and it is likely that soon these techniques will be employed extensively in genetics research and across multiple fields of medicine.

## Figures and Tables

**Table 1:** Gene therapy trials for retinal diseases, with relevant articles cited.

Disease	NCT ID & Sponsor	Phase	Intervention	Citations
**Leber Congenital Amaurosis**	NCT00999609 NCT01208389 NCT00516477 Spark Therapeutics (Philadelphia, PA, USA)	III I, II I	Subretinal AAV2-hRPE65v2	[7,29,30,31,32,33]
	NCT00643747 University College, London (London, UK)	I, II	Subretinal rAAV2/2.hRPE65p.hRPE65 (tgAAG76)	[5,34,35,36]
	NCT00749957 Applied Genetic Technologies Corp. (Alachua, FL, USA)	I, II	Subretinal rAAV2-CB-hRPE65	[37]
	NCT01496040 Nantes University Hospital (Nantes, France)	I, II	Subretinal rAAV2/4.hRPE65	
	NCT02781480 University College, London (London, UK)	I	Subretinal AAV2/5 OPTIRPE65	[38]
	NCT00481546 University of Pennsylvania (Philadelphia, PA, USA)	I	Subretinal rAAV2-CBSB-hRPE65	[39,40,41,42]
**Choroideremia**	NCT02077361 Ian M. MacDonald	I, II	Subretinal rAAV2.REP1	
	NCT02553135 Byron Lam	II	Subretinal AAV2-REP1	
	NCT01461213 NightstaRx (London, UK)	I, II	Subretinal AAV2.REP1	[43,44,45]
	NCT02671539 STZ Eyetrial (Tübingen, Germany)	II	Subretinal rAAV2.REP1	
	NCT02341807 Spark Therapeutics (Philadelphia, PA, USA)	I, II	Subretinal AAV2-hCHM	
**Achromatopsia**	NCT02599922 Applied Genetic Technologies Corp. (Alachua, FL, USA)	I, II	Subretinal rAAV2tYF-PR1.7-hCNGB3	[46,47,48]
	NCT02610582 STZ eyetrial (Tübingen, Germany)	I, II	Subretinal rAAV.hCNGA3	
**X-linked Retinoschisis**	NCT02317887 National Eye Institute (Bethesda, MD, USA)	I, II	Intravitreal AAV8-scRS/IRBPhRS	[49,50,51,52]
	NCT02416622 Applied Genetic Technologies Corp. (Alachua, FL, USA)	I, II	Intravitreal rAAV2tYF-CB-hRS1	[53,54]
**Leber Hereditary Optic Neuropathy**	NCT02652767 NCT02652780 GenSight Biologics (Paris, France)	III III	Intravitreal GS010; Sham intravitreal injection	[55]
	NCT02161380 John Guy	I	Intravitreal scAAV2-P1ND4v2	[56,57,58]
	NCT01267422 Bin Li	--	Intravitreal rAAV2-ND4	[59]
**Retinitis Pigmentosa**	NCT02556736 Retrosense Therapeutics (Ann Arbor, MI, USA)	I, II	Intravitreal RST-001	[60,61]
	NCT01482195 Fowzan Alkuraya	I	Subretinal rAAV2-VMD2-hMERTK	[62,63]
**Usher Syndrome**	NCT01505062 NCT02065011 Sanofi (Paris, France)	I, II I, II	Subretinal EIAV-CMV-MYO7A (UshStat)	[64]
**Stargardt Disease**	NCT01736592 NCT01367444 Sanofi (Paris, France)	I, II I, II	Subretinal SAR422459	[65,66,67]
**Neovascular Age-Related** **Macular Degeneration**	NCT01494805 Lions Eye Institute (Perth, WA, Australia)	I, II	Subretinal rAAV.sFlt-1; Control (ranibizumab alone)	[68,69,70]
	NCT01024998 Genzyme, a Sanofi company (Cambridge, MA, USA)	I	Intravitreal AAV2-sFLT01	[71,72,73]
	NCT01301443 NCT01678872 Oxford BioMedica (Oxford, UK)	I I	Subretinal RetinoStat	[73,74,75]

**Table 2:** Clinical trials for natural disease characterization of retinal degenerations.

	NCT ID & Sponsor	Design (Gene of Interest)
**Leber Congenital Amaurosis**	NCT02575430 QLT Inc. (Vancouver, BC, Canada)	Retrospective (*LRAT* & *RPE65)*
	NCT02714816 MeiraGTx UK II Ltd. (New York, NY, USA)	Prospective (*RPE65*)
	NCT00422721 Nantes University Hospital (Nantes, France)	Prospective (*RPE65*)
**Choroideremia**	NCT02994368 4D Molecular Therapeutics (Emeryville, CA, USA)	Prospective (REP-1)
**Achromatopsia**	NCT01846052 Applied Genetic Technologies Corp. (Alachua, FL, USA)	Prospective (*CNGB3*)
**Leber Hereditary Optic Neuropathy**	NCT02796274 Santhera Pharmaceuticals (Liestal, Switzerland)	Retrospective (G11778A, G3460A, and T14484C mtDNA mutations)
**X-linked Retinoschisis**	NCT00055029 National Eye Institute (Bethesda, MD, USA)	Prospective (*RS1*)
**Retinitis Pigmentosa**	NCT02759952 STZ Eyetrial (Tübingen, Germany)	Prospective (*PDE6A*)
	NCT01021982 Sheba Medical Center (Ramat Gan, Israel)	Prospective
	NCT01949623 Johns Hopkins University (Baltimore, MD, USA)	Prospective biomarker study
	NCT02926092 Shire (Dublin, Ireland)	Prospective (*RHO*)
	NCT00784901 National Taiwan University Hospital (Taipei City, Taiwan)	Retrospective
**Usher Syndrome**	NCT00106743 National Eye Institute (Bethesda, MD, USA)	Prospective
**Stargardt Disease**	NCT01977846 Foundation Fighting Blindness Clinical Research Institute (Columbia, MD, USA)	Retrospective & Prospective (*ABCA4*)
	NCT02410122 John Hopkins University (Baltimore, MD, USA)	Prospective (*PROM1*)
	NCT01736293 National Eye Institute (Bethesda, MD, USA)	Prospective (*ABCA4*)
